# Impact of the COVID-19 pandemic on the sexual and reproductive health of adolescents in Alberta, Canada

**DOI:** 10.1186/s12978-023-01712-x

**Published:** 2023-11-22

**Authors:** Salima Meherali, Amyna Ismail Rehmani, Mariam Ahmad, Bisi Adewale, Samar Kauser, Simone Lebeuf, James Benoit, Shannon D. Scott

**Affiliations:** 1 Faculty of Nursing, University of Alberta, Edmonton Clinic Health Academy, 11405 87 Avenue, Edmonton, AB T6G 1C9 Canada; 2 Department of Pediatrics, University of Alberta, Edmonton Clinic Health Academy, 11405-87 Avenue, Edmonton, AB T6G 1C9 Canada

**Keywords:** Adolescents, Sexual and reproductive health (SRH), COVID-19 pandemic, Service providers, Canada

## Abstract

**Purpose:**

The COVID-19 pandemic led to major service disruptions in the healthcare sector, especially regarding sexual and reproductive health services. However, the impact of the pandemic on Canadian adolescents is relatively unknown. This study aimed to investigate the impacts of the COVID-19 pandemic and associated public health measures on the sexual and reproductive health (SRH) of adolescents in Alberta, Canada.

**Methods:**

A qualitative study using an interpretive description (ID) approach and community-based participatory research principles was conducted to capture the subjective experience and perceptions of adolescents and service providers. With the collaboration of the Adolescent Advisory Group and community partners, 18 adolescents and 15 service providers were recruited for the study through purposive sampling. Findings from the qualitative interviews were analyzed using thematic analysis.

**Results:**

Three major themes emerged from the analysis: (1) COVID-19 SRH experience, (2) barriers to SRH, and (3) adolescent SRH strategies. Our findings highlight numerous barriers and challenges that prevented adolescents from accessing SRH education, products, and services.

**Conclusion:**

The COVID-19 pandemic had a profound impact on the SRH and the well-being of adolescents. Our study reflects the need for diverse SRH strategies to maintain continued access to SRH resources during disruptive events, such as the pandemic.

**Supplementary Information:**

The online version contains supplementary material available at 10.1186/s12978-023-01712-x.

## Plain language summary

Access to sexual and reproductive health (SRH) services is a basic human right. All individuals require access to appropriate SRH services to maintain their optimal sexual and reproductive health. Adolescents require special guidance, support, and youth-friendly services in matters of SRH as they enter puberty and explore their sexual identity. However, during the COVID-19 pandemic, many health and SRH services were suspended. Access to SRH products and services became difficult due to public health restrictions, which has possible negative consequences for adolescents’ SRH. The experiences of adolescents during the COVID-19 pandemic regarding their SRH are not reported in Alberta, Canada. Therefore, we explored the impacts of these public health restrictions on adolescents’ SRH. We performed qualitative interviews with adolescents and SRH service providers to know their perspectives on how the pandemic influenced the SRH of adolescents. This paper provides insights into the barriers faced by adolescents while accessing SRH services during the pandemic, as well as their perceptions of digital strategies, such as mobile applications, and other recommendations for supporting SRH education and services. Based on the study findings, an adolescent-friendly mobile application will be developed to provide a virtual platform connecting adolescents to SRH educational resources, services, and support.

**Supplementary Information:**

The online version contains supplementary material available at 10.1186/s12978-023-01712-x.

## Background

Globally, the COVID-19 pandemic led to drastic changes in the access and delivery of healthcare services, influencing the physical, psychological, social, and emotional well-being of individuals, and profoundly impacted adolescents [1, 2]. The World Health Organization (WHO) defines adolescence (ages 10–19) as a period of rapid physical, cognitive, and psychosocial growth [3], marked by the onset of puberty and sexual maturity [4]. This period necessitates care, services, and education regarding sexual and reproductive health and rights (SRHR), as well as preventing negative consequences to SRH (e.g., sexually transmitted infections, and unintended pregnancy) [5–7].

A Canadian Community Health Survey (2015/2016) reported that more than half (54.1%) of 15–24-year-olds in Canada were sexually active and at risk of unintended pregnancy and/or STIs [8]. Adolescents in Canada give birth to close to 13,000 newborns each year [9]. Adolescent mothers are at increased risk of mental health problems, substance use disorders, domestic violence, STIs and recurrent pregnancies, which are often associated with lower socio-economic status, poverty, and lack of social support [9, 10]. Wong et al. [11] found that youth experiencing economic or social marginalization were more likely to have poorer SRH outcomes.

Adolescents often experience a range of intersecting barriers making it difficult to access appropriate SRH services (e.g., low socio-economic status, stigmas, lack of culturally safe services) [6, 12]. The pandemic imposed additional barriers further reducing access to SRH services. Prioritization of resources for essential COVID-19 response over SRH services resulted in the closure of safe abortion sites, reduced supply of contraceptives, and suspension of SRH services. As a result, this is expected to increase cases of gender-based violence (GBV), unplanned pregnancies, unsafe abortions, and maternal mortalities [2, 13, 14]. The closure of schools and SRH clinics limited opportunities for adolescents to gain comprehensive SRH education and utilize SRH services or products [15, 16]. Economic constraints, loss of income, and limited health insurance coverage during the pandemic further impeded seeking SRH care, undergoing STI testing/treatment, or purchasing contraceptives [7, 17].

The impacts of the pandemic are still unfolding and there are potential long-term consequences that will shape adolescents’ SRH. However, the impacts of these measures on the SRH of adolescents in Alberta, Canada is relatively unknown. Understanding the effects of these measures is a crucial step towards informing effective services, supports, and strategies to improve adolescents’ access to SRH services. Therefore, the overall purpose of our study was to investigate the impact of the COVID-19 pandemic and associated public health measures on the SRH of adolescents in Alberta. Specifically, the study addressed three research questions:

1. What are the perspectives of adolescents and service providers on how the pandemic has influenced the SRH of adolescents in Alberta?
2. How did pandemic-related public health measures shape access to SRH services by adolescents?
3. What types of services, programs, or supports for SRH did adolescents use during the COVID-19 pandemic?

## Methods

### Theoretical framework

We used Levesque’s conceptual framework of access to health care [18] to understand and examine adolescents’ access to SRH services during the pandemic. It provides a comprehensive and multidimensional view of healthcare access in the context of the healthcare system with dimensions of approachability, acceptability, availability/accommodation, affordability, and appropriateness, along with considering the population’s socioeconomic determinants and abilities to perceive, to seek, to reach, to pay, and to engage, in healthcare [18, 19]. We utilized these principles to understand adolescents’ access from both provider (supply) and adolescent (demand) dimensions.

### Study design

We conducted this study in two phases over 2 years (2021–2023). The first phase of the study consisted of qualitative interviews with adolescents and care providers, whereas the second phase consisted of co-designing an adolescent specific SRH website based on the knowledge generated from the interviews. In this manuscript, we are presenting the findings from the qualitative interviews.

#### Phase 1: qualitative interviews

To enhance the integration of research findings into practice, programming, and policy and to promote the co-creation of knowledge within the study, we used an interpretive description (ID) approach combined with integrated knowledge translation (IKT) [20]. ID facilitates knowledge development by capturing the subjective experience and perception of the group under study advancing the understanding of the phenomenon under study [21]. We applied the principles of ID in our study through purposeful sampling, concurrent data collection and analysis, and ensuring rigour (establishing methodological coherence, verification of coding system, field notes etc.) [20]. An Adolescent Advisory Group (AAG), pre-established by SM (Principal Investigator) in Edmonton, was engaged in this study to contribute to the recruitment process, data collection and development of KT activities (Table 1).

**Table 1:** Demographic characteristics of adolescent advisory group (n = 12)

Characteristics	*N* (%)
*Gender*	
Female	8 (67)
Male	4 (33)
*Age*	
17 years	4 (33)
18 years	6 (50)
19 years	2 (17)
*Ethnicity*	
Lebanese	2 (17)
Egyptian	1 (1)
Somalian	1 (1)
Indian	4 (33)
Pakistani	2 (17)
Caucasian	2 (17)
*Sexual orientation*	
2SLGBTQIA+	2 (17)
Heterosexual	10 (83)
*Gender identity*	
Cisgender	12 (100)

### Participants and recruitment

We used a multi-faced community-based strategy to recruit adolescents, which involved social-media marketing campaigns, university-wide weekly email campaigns, and promotion of the study in the university campus and clinics. In collaboration with our community partners who provide care to vulnerable youth, we recruited participants from various backgrounds, socioeconomic standing, and gender identities to ensure adequate representation. We also recruited SRH service providers through Adolescent Medicine Clinic and Birth Control Centre in Edmonton.

Participants were included in the study based on the following inclusion criteria: (1) Adolescents aged 15–19 years old who were seeking or had accessed SRH services in the past 5 years or healthcare professionals who provided SRH care to youth, (2) living in Alberta, and (3) fluent in English. We included older adolescents (15–19 years) in this study as their SRH needs are different from younger adolescents (10–14 years).

We used purposive sampling to deliberately choose participants based on the inclusion criteria, who would provide relevant and rich descriptions of their SRH experiences. Informed consent was obtained from all participants and additional parental consent was obtained if it was determined that adolescents lacked decision-making capacity. A total of 18 adolescents and 15 healthcare providers were recruited for the study. This sample size was determined a priori and deemed adequate to gain a deeper understanding of participant viewpoints while acknowledging that there may be differences in perceptions and outliers [20]. Due to COVID-19 restrictions, all recruitment and subsequent data collection occurred online via E-mail and Zoom between November 2021–January 2022.

### Data collection

Data collection was conducted from 2021 to 2022. Ethics approval was obtained from the University Research Ethics Board. Data were collected through a semi-structured interview guide (Additional file 1, Additional file 2), which was co-developed with the AAG, and included open-ended questions on the impact of COVID-19 on everyday life, SRH, access to services, and sources of support. Interviews were conducted by SM with expertise in qualitative methods and SRH of adolescents along with a trained research assistant. Interviews lasted for 30–60 min and were audio-recorded through Zoom. Audio recordings were transcribed to remove all identifying information. The demographic data of participants were collected through secure Google forms.

### Data analysis

Conforming to ID methods, we performed data collection and analysis concurrently through an iterative process [20]. Data analysis was conducted in four stages: (1) Audio recordings (recorded via Zoom) were transcribed verbatim, (2) research assistants (RA) read transcripts multiple times and double-checked for accuracy, (3) SM and RA conducted open coding and grouped codes into preliminary themes using thematic analysis, (4) themes across the interviews were grouped into an organizational framework. Qualitative analysis software NVivo version 12 (Denver, Colorado) was used to analyze and code data from both adolescents and service provider interviews.

### Rigour

To achieve reliability and validity, we employed verification strategies to identify when to continue, stop, or modify the research process. Verification strategies included: (1) Methodological coherence; ensured congruence between the research questions and components of ID, (2) appropriate and diverse sampling to ensure efficient and effective saturation of categories with optimal quality, (3) collected and analyzed data concurrently, (4) developed a coding system that was discussed and verified with research team members including AAG, (5) recorded field notes and evaluated the quality of the interviews consistently by analyzing the participant’s responses to the questions and modifying the questions accordingly.

## Results

### Participant characteristics

See Tables 2, 3.

**Table 2:** Demographic characteristics of adolescents (n = 18)

Characteristics	*n* (%)
*Gender*	
Female	9 (50)
Male	9 (50)
*Age*	
14–15 years	1 (5.5)
16–17 years	5 (27.7)
18–19 years	12 (66.6)
*Place of birth*	
Canada	12 (66.6)
Pakistan	2 (11.1)
India	3 (16.6)
Venezuela	1 (5.5)
*Number of years in Canada*	
Less than 1 year	1 (5.5)
1 to 3 years	1 (5.5)
More than 10 years	16 (88.8)
*Level of education*	
Middle school	1 (5.5)
High school	6 (33.3)
Post-secondary education	11 (61.1)
*Languages spoken at home (multiple responses)*	
English	16 (88.8)
French	2 (11.1)
Spanish	1 (5.5)
Urdu	2 (11.1)
Hindi	4 (22.2)
Punjabi	1 (5.5)
Arabic	2 (11.1)
Gujarati	2 (11.1)
Others	2 (11.1)
*Place of birth (parents)*	
Mother	
Canada	10 (55.5)
India	4 (22.2)
Pakistan	1 (5.5)
South Korea	1 (5.5)
Venezuela	1 (5.5)
Iran	1 (5.5)
Father	
Canada	8 (44.4)
Pakistan	2 (11.1)
France	1 (5.5)
Venezuela	1 (5.5)
India	4 (22.2)
Nigeria	1 (5.5)
Libya	1 (5.5)
*Parents’ level of education*	
Mother	
College	5 (27.7)
University	13 (72.2)
Father	
High school	4 (22.2)
College	3 (16.6)
University	11 (61.1)

**Table 3:** Characteristics of service providers (n = 15)

Characteristics	*n *(%)
*Healthcare role*	
Public Health Nurse	6 (40)
Partner Notification Nurse	3 (20)
Health Educator (RN)	2 (13.3)
Intake Coordinator (RN)	4 (26.6)
*Location*	
Edmonton	7 (46.6)
Calgary	4 (26.6)
Fort McMurray	3 (20)
Medicine Hat	1 (6.6)
*Years of experience*	
More than 20 years	2 (13.3)
11–20 years	3 (20)
6–10 years	7 (46.6)
0–5 years	3 (20)

### Key findings

Three broad themes emerged from the adolescents’ and service providers' qualitative interviews: (1) COVID-19 SRH experience, (2) barriers to SRH, and (3) adolescent SRH service access strategies. These themes were then further divided into sub-themes, which are presented below.

#### 1) COVID-19 SRH experience

The COVID-19 pandemic influenced many institutions including adolescents' access to sexual and reproductive health. We found that adolescents had both positive and negative experiences during the pandemic.

##### Impact of the COVID-19 pandemic

Many adolescents reported both positive and negative changes in their SRH services before and during the pandemic. These changes were associated with the challenges of accessing walk-in clinics and longer times to seek SRH services:

…It was definitely a lot harder to find that kind of access as well because of I assume, less staff working and more pandemic measures, so, while I assume it should have taken about a week it took a month to get the service that we did. (Adolescent Participant-1)

Service providers commented on the de-prioritization of SRH services during the pandemic: "I think this kind of got pushed aside – like sexual health like wasn’t people’s priority, of course, with COVID going on." (Health Educator).

Another service provider also mentioned:

Definitely not having the walk-in impacted youth, not providing the open access, you know, all the screening tools, the mask. It just became a whole ordeal just to even come in and pick up their birth control or, you know, get emergency contraceptives, or get tested. It was, you know, a whole lot, where it was just before, it was easy. They’d just come, they could go. So definitely, our access, and the walk-in, not having that available for them was probably that. (Public Health Nurse)

As consultation moved from in-person to online, some adolescents expressed their dissatisfaction with online services, especially with matters regarding counselling and family planning. They reported feeling uncomfortable with a healthcare provider in a virtual setting:

I needed to talk to my doctor about something and I wanted her to like look at something or like have a visual she only does phone appointments now most of the time, and then, if she didn't do for an appointment it's like she's only in office, I think, like two days a week, and that's changed definitely because she used to be an office I think. (AP-9)

Alternatively, many adolescents indicated online services to be a positive outcome of the pandemic: “Discussing with my doctor over the phone was very efficient and faster than it would have been in person” (AP-14).

Some participants stated that there was no impact of the pandemic on their SRH: “Personally, no. I don’t think it’s done anything like about my reproductive health, really. Like before and after, or even during, it was nothing’s really changed.” (AP-11).

##### Availability and accessibility of SRH products and services

Several adolescents reported the easy accessibility of using alternate (online) services for their SRH needs and highlighted the use of newer resources like mobile applications to support their SRH. Although some adolescents appreciated the online services, many struggled with lengthy waiting times and securing SRH products (contraceptives). They recalled the ease of obtaining free contraceptives and medical advice from clinics on a walk-in basis before the pandemic: “It’s also a lot harder to find free like birth control, like condoms or whatever versus before I just would pick them up in my local clinic.” (AP-4).

Another participant also expressed:

There's definitely a decrease in in-person services and I also think that there's much more of a wait time when it comes to picking up stuff like birth control or asking basic questions that I could easily have asked my physician before the pandemic. (AP-10)

Service providers talked about the challenges that were introduced in the system during the pandemic: "During the worst of the pandemic, there was only one nurse. It was by appointment only, which kids don’t do great with appointments. And it was symptomatic testing only." (Intake Coordinator).

Additionally, workforce shortages because of redeployment to COVID-19 response further compromised SRH operations:

So, staff shortages were – were typically because of redeployment to COVID-19 efforts, and so when your staff is doing something else, then you have limited access in terms of all of it (Public Health Nurse)

#### 2) Barriers to SRH

We explored the barriers that adolescents encountered during the pandemic as many challenges were brought on by the closure of schools, limited hours of stores, and the absence of support systems.

##### Reduced SRH education through schools

Many adolescents indicated that the majority of their SRH education was obtained through the school. Formal classes on sexual education (sex-ed), as well as other courses, helped them to gain an understanding of their bodies and SRH, which got interrupted. As participant 14 mentioned: “I mean most of my information has come from school resources such as reproductive health classes and biology classes, so that is my main means for understanding reproductive and sexual [health].”

Another participant expressed:

During the pandemic, you know, due to the closure of schools, it affected the information I used to get, and you know, libraries. Like in most cases, I used to go to the library to, you know, have access to library materials to read more about sexual education and reproduction. (AP-12)

A service provider commented on how the pandemic led to the suspension of SRH outreach education initiatives:

So, before the pandemic, our clinic did a lot of outreach, so we’d go to like – it’s at the high school for kids that struggle in regular high school. We would go there. We would go to other little places around the community, and then with COVID, that obviously stopped. (Health Educator)

During the later stages of the pandemic when educational institutions switched to online platforms, SRH education was not given a higher priority:

It definitely like affected it, because I missed out on my grade ten and eleven – well, sorry, eleven and half of twelve school year, so it was like – like that’s like prime age for kids to start like exploring, and like I didn’t get that sex ed during those years, so like it was definitely more of a figure it out yourself kind of thing that happened, to like the age group that I belonged in, because we didn’t get taught it, because it wasn’t a priority, so they didn’t teach it over Zoom. (AP-16)

##### Limited store hours

Participants reported that reduced hours of stores during the pandemic decreased access to SRH products, like contraceptives and menstrual hygiene products: “I guess during the pandemic it definitely got a little bit harder, because of like the mandates and stuff like that, some stores were closed (AP-11).

Another participant expressed:

The ability to go to the store became a little bit harder because of limited capacities and things a lot of stores have only 25% capacity or 30% capacity, so it was hard just having to like wait in lines and things but that's just like a general inconvenience. (AP-17)

Another participant reported difficulties securing contraceptives: “Like if I was getting contraceptives, it was definitely harder to get during the pandemic because stores were closed down.” (AP-11).

Reduced supply of SRH products during the pandemic, such as contraceptives was also reported: “Lower store capacities or stock of items, especially when like toilet paper and, like other hygiene products things became a little bit harder.” (AP-17).

##### Lack of social support

Many adolescents expressed the loss of social support and connectedness during the pandemic. Social distancing mandates made it difficult to meet peers and share common concerns:

Like it's a lot harder to find a group like I said and to find people that you can rely on to support you as well, I felt like it was like I was discouraged during the beginning of the pandemic and during peak or like when there was more cases to go and get tested. (AP-8)

Another participant talked about losing other forms of support, such as counselling: “Yeah, kind of both, because the only counselling I would have had access to would have been school, and when schools went online, there was really no way to do that.” (AP-16).

One participant expressed the difficult journey of exploring their sexual orientation during the pandemic without any support:

So I found that has really impacted me and my sexual and reproductive health, during the pandemic I can't really talk about that, before because I don't think I would have gone through the journey that I've gone through with my sexuality if it wasn't for this pandemic but I do know that resources have been a lot harder for LGBTQ+ individuals during this time. (AP-4)

#### 3) Adolescent SRH service access strategies

We inquired about adolescents’ perceptions of the development of a mobile application to access SRH resources and what strategies proved to be useful to them during the pandemic. Participants recommended strategies that they believed would be more beneficial for the adolescents.

##### Outlook on SRH digital strategies

The majority of the participants in this study supported the idea of developing a mobile application for SRH resources and indicated that it would increase access to SRH resources. One participant stated their preference for mobile applications over other digital platforms:

Yeah, I definitely would recommend that (mobile applications) I think would be really useful, especially because I'm on websites sometimes the university website sometimes just in general websites on the Internet, it can be hard to navigate them like they aren't made in a way that it's easy to know where things are located. (AP-1)

Service providers shared their experiences of using digital strategies for SRH education:

Like there’s actually a lot of good technology out there that I knew about and could use. Most of the time it was tech problems on the school side. Like there was lots of problems getting – if there was two classes, getting both teachers to log in, because the person who made the Google lead had to let them in. I couldn’t let them in. So, like there was like those little issues. (Health Educator)

On one hand, participants expressed that mobile applications would provide adolescents with the privacy to seek SRH help confidentially: “That's really cool and it could be super useful, especially for like people who are in families that don't feel comfortable talking about those things are no” (AP-13). On the other hand, some had concerns regarding the safety features of such applications and the possibility of receiving unreliable information:

Oh yes, and mobile apps I tend to be a bit more wary because there is a lot of misinformation and bad apps, I guess, but the online websites if it looks credible than I do, listen to what they say and they have helped me get more information on productive health and, what is needed to ensure that I'm being healthy and safe. (AP-14)

##### Supporting SRH through various strategies

We explored various strategies and recommendations from participants that would help in developing targeted interventions to support adolescents’ SRH. Participants talked about various features to facilitate the delivery of SRH resources and provide a holistic platform for adolescents to receive support. One participant indicated the importance of a simple interface design:

Maybe simplifying the online website would be helpful, so making the interface easier to navigate and having more information summarized - because sometimes some aspects aren't very clearly explained so just summarizing where things and having an easier interface would be very helpful. (AP-14)

Another participant recommended anonymous forums where adolescents with similar interests can freely discuss SRH with care providers:

Just a mobile app that makes information easy to consume because some people are like shy to look things up and just some maybe anonymous forums like so we can have this community of adolescents, to discuss freely about their problems rather than being embarrassed by having their public identity revealed so just some forums and some information and maybe some clinicians, if possible. (AP-7)

Service providers highlighted how they supported adolescents’ SRH by implementing various strategies:

I did a lot of texting with our youth patients during the pandemic. And I found that that was a really helpful way to sort of deliver a lot of information at once delivered in a way that's like immediate and accessible to the youth. (Public Health Nurse)

Along with videos, Zoom, like virtual sessions were also effective, because again, I can reach more rural. And we made a frequently asked questions PDF because usually we get really similar questions... And so that was really helpful because they can just read that and get their questions answered. (Partner Notification Nurse)

Many participants also expressed seeing various features to help them get quick access to resources and nearby SRH services:

I think that an app or like a website with like STI testing centers or different clinics and areas that you can go and get you know condoms birth control, whatever we can get all of this help just one app or one website that has all of that information there with very minimal searching. I think that would be something that would really benefit society because it's very hard to find information. (AP-4)

One service provider indicated how virtual strategies can target adolescents effectively and reduce barriers to their SRH and well-being:

I think like it’s the younger generations that are really doing well with like the virtual appointments. I think if they could find a way to make access to that more easier, I think kids would be more comfortable getting tested, instead of having to – you know, it’s intimidating for a 15 or 16-year-old kid to walk into a health building all by themselves, try to register themselves, sign in, go to the right desk, answer all these medical questions. So, it’d be nice if there was a more comfortable environment for them, perhaps. (Public Health Nurse)

## Discussion

This study evaluated the impact of the COVID-19 pandemic on the sexual and reproductive health of adolescents and explored the factors that supported and promoted their SRH. We found that the COVID-19 pandemic had significant impacts on various aspects of the SRH of adolescents, including but not limited to access to SRH education, services, and products.

**Fig. 1:**
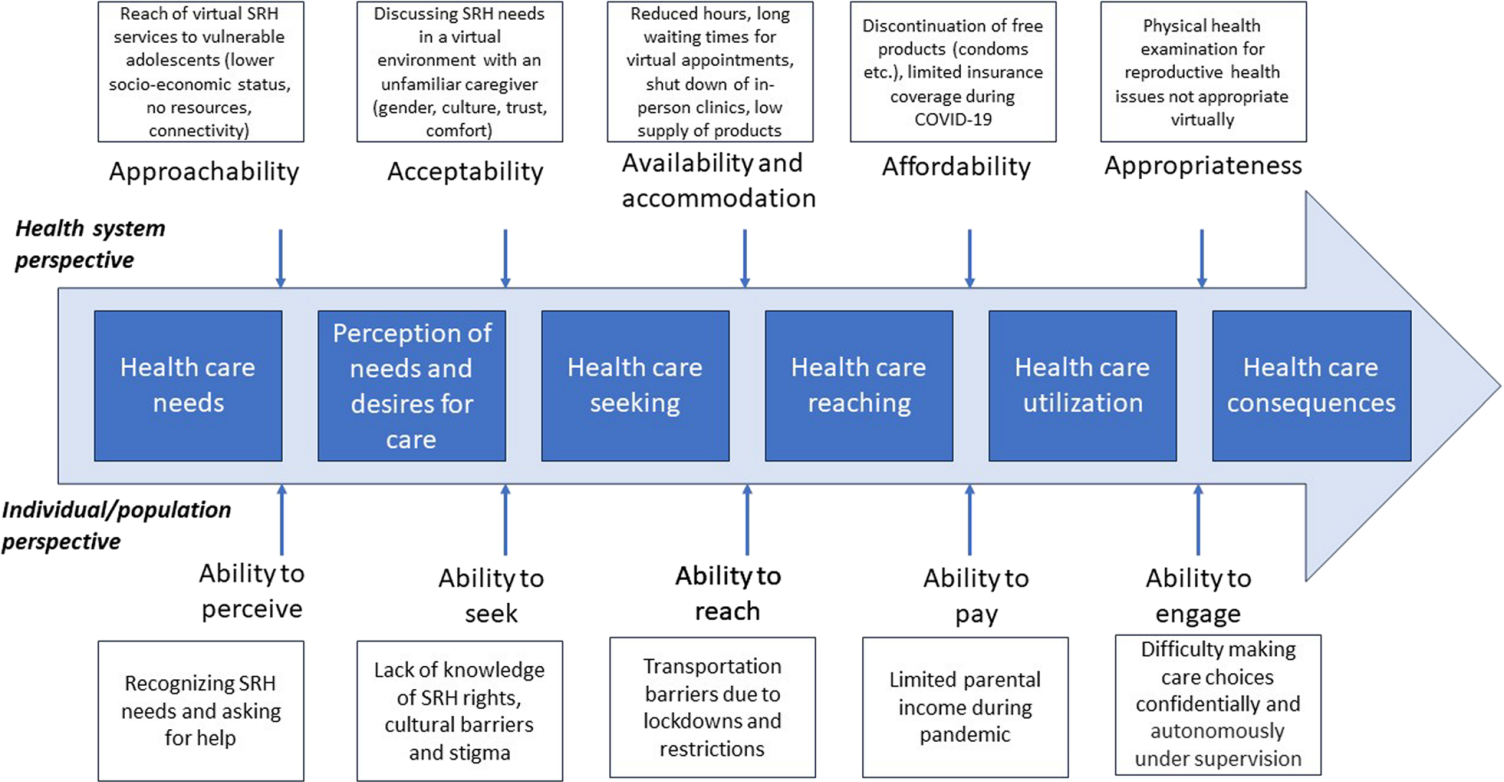
Adaptation of Levesque’s conceptual framework for healthcare access [18]

The pandemic disrupted major sectors, including preventive healthcare, leading to the loss of basic SRH rights [21]. Diverting resources to the acute crisis and restricting other services were necessary, but prioritizing the SRH needs of individuals was also important as the pandemic did not halt those needs. In fact, those needs increased due to the additional barriers (e.g., reduced clinic hours and limited in-person patient intake, transportation, lack of affordable SRH products etc.) introduced in the existing system [22, 23]. We used Levesque’s conceptual framework of access to health care to understand and categorize the barriers that adolescents encountered while accessing SRH care during the pandemic, both from the healthcare system’s perspective and individual perspective. Figure 1 presents the adaptation of the conceptual framework to highlight those barriers and challenges in the dimensions of approachability, acceptability, availability and accommodation, affordability, and appropriateness along with the individual factors that impact access to care [18, 19]. Adolescents in our study reported interruptions in SRH services during the pandemic which affected their physical, emotional, sexual and mental health. These findings are consistent with several other studies globally where SRH services were partially suspended and deemed non-essential during the pandemic [21, 24–26].

Adolescents in our study reported the challenges of securing SRH products timely (i.e., contraceptives, condoms), which was brought on by the closure of schools, stores, and in-person clinics. The unavailability of contraceptives (i.e., manufacturing and logistical delays) [7] and in-person clinics reduced their ability to access SRH products and services. Despite the barriers, adolescents were found to be sexually active during the pandemic and therefore engaged in risky and unprotected sexual behaviours, leading to potential unpredicted outcomes (i.e., pregnancy, STIs) [23, 24]. Maier et al. [25] reported that several states in the U.S. suspended essential SRH services during the pandemic, especially abortion services, failing to satisfy the basic SRHR of individuals. Although not evident from our findings, the literature from low—middle—income countries suggest an increase in the number of unintended pregnancies and STIs among adolescents during the pandemic due to these service disruptions [13, 23]. A study in the U.K. highlighted the disproportionate impact of SRH service suspension on adolescents as contraception and STI screening became less accessible and available [27]. However, instead of completely suspending essential SRH services during lockdowns, such as abortion care, alternative approaches were used to deliver SRH care through remote appointments and medical abortion (abortion pills) treatment packages delivered on request [27, 28].

Adolescents faced a major setback when physical distancing requirements prevented them from meeting their social circles and receiving support from their peers, counsellors or service providers. Loss of support systems and educational and extracurricular activities increased anxiety among adolescents, leading to increased risky behaviours, including unprotected sexual activity [23]. A few adolescents in our study expressed how all these factors eventually impacted their mental health. Magson et al. [29] reported longitudinal declines in the mental health of adolescents during the pandemic which were associated with the loss of social connectedness and increased parental conflicts.

The shift from in-person to online SRH services (i.e., telemedicine) was well appreciated by many adolescents in this study, as it not only prevented longer waiting times in high-risk COVID-19 environments but also made services accessible at the fingertips. Service providers also reported that online services improved access and gave them an opportunity to connect with adolescents. Regardless of the ease of access, some participants expressed their discomfort and dissatisfaction with meeting healthcare providers in a virtual setting. Although telemedicine created ease for some SRH issues, many other reproductive health issues necessitated physical examinations (e.g., pelvic inflammatory disease) or laboratory investigations (e.g., STI screening, treatment) [30]. Our findings align with another study where adolescents expressed a lack of confidence while communicating with care providers online independently and confidentially [24].

The pandemic caused a massive shift from in-person SRH care to self-management through telemedicine and digital resources [13]. Adolescents in our study utilized digital SRH resources to access SRH information. They also communicated the need for adolescent friendly SRH websites and mobile applications where they can receive comprehensive SRH resources altogether. Mobile phone supported SRH interventions are needed as they have immense potential to offer safe, confidential, accurate, and timely information and services to adolescents [31], and can increase accessibility by making services more approachable, affordable, and available [18]. Regardless, there are some limitations (e.g., internet access in remote areas, technical difficulties) that may restrict the acceptability and appropriateness of SRH digital interventions [18]. Our future efforts will focus on co-designing an adolescent friendly SRH website and mobile application to improve SRH access to adolescents, especially those who are at more risk of SRH inequalities.

### Strengths and limitations

This study is the first to report the experiences of adolescents regarding their SRH during the COVID-19 pandemic in Alberta, Canada. We collected and analyzed data from both adolescents and service providers, which yielded a comprehensive understanding of the challenges faced by adolescents. Also, we recruited participants from various backgrounds to ensure a better representation of the sample.

Due to the sample size and geographical limitations, the results may not be generalizable to the entire population. Data were collected only from Alberta, and the results could vary in other provinces and territories. We did inquire about adolescents’ current level of education but did not inquire about their school system (e.g., private/public) as this could highlight the similarities or differences in the provision of SRH education across education systems. We did not collect data on participants’ level of sexual activity or contraceptive methods; this can be beneficial in future to determine the impact of public health restrictions on sexual activity and/or unintended pregnancies. While our study focused on the SRH experiences of adolescents aged 15–19 years, we acknowledge the limitation that some participants might have referenced SRH services accessed as early as age 10, and we did not consistently specify the exact age or nature of the service at the time of their experiences. Studies in the future can address the nature of SRH services accessed in different age groups. Also, we did not analyze the impact of the pandemic in relation to the socio-demographic characteristics of the participants, which could provide multi-dimensional perspectives on SRH experiences. Future research can explore using the principles of intersectionality and health equity in determining the SRH experiences of adolescents during disruptive events.

## Conclusion

In conclusion, the COVID-19 pandemic had a profound impact on adolescents' access to SRH education and services. School closures, limited store hours, and a lack of social support contributed to an overall decline in SRH well-being. Adolescents faced numerous barriers in obtaining essential SRH products and services and experienced a knowledge gap because of the pandemic. Service providers also experienced barriers in reaching adolescents for SRH education and services during the pandemic. Most participants supported the development of mobile applications as they can increase access to SRH resources. By incorporating these insights, targeted interventions can be developed to enhance SRH well-being and empower adolescents to navigate their SRH confidently during public health crises. Future research and policy efforts should focus on developing strategies that accommodate both in-person and remote services, ensuring that adolescents can access SRH services according to their needs and preferences, and fostering social support networks for adolescents in times of crisis.

### Supplementary Information

**Additional file 1.** Interview guide for adolescents.

**Additional file 2.** Interview guide for service providers.

## Data Availability

All data generated or analyzed during this study are included in this published article.
