# Supplementary material for: Impact of the COVID-19 pandemic on the sexual and reproductive health of adolescents in Alberta, Canada
Source: Reprod Health. 2023 Nov 22;20:172. doi: 10.1186/s12978-023-01712-x (PMC10664423; doi:10.1186/s12978-023-01712-x)
Supplement: Supplementary file 1 — Additional file 1. Interview guide for adolescents. [file 12978_2023_1712_MOESM1_ESM.docx]

**Interview Guide for Adolescents (draft)**

**Impact of the COVID-19 Pandemic on Sexual and Reproductive Health of Adolescents in Alberta**

Initial Open-Ended Questions

1. Tell me briefly about your SRH needs before and during the COVID-19 pandemic? (Access to SRH education, provision of contraceptive, prevention and treatment of HIV, and other STIs, abortion care, antenatal or postnatal care, prevention and care response to sexual and gender-based violence, counselling and services for sexual health and well-being, including provision of menstrual health information and services).
2. Do you think pandemic has impacted your SRH? How?
3. How has the pandemic influenced your access to SRH services?
4. What are the challenges you have faced in receiving SRH services during the COVID-19 pandemic? [Probe about privacy, access to in-person services and walk-in service etc.]
5. Does school closure impact your access to SRH services?
6. How long do you have to wait to get support for SHR services during the pandemic compared to before the pandemic?
7. What types of services, programs, or supports for SRH services did you use during the COVID-19 pandemic?
8. Are you able to access SRH services through other means apart from going in person to the clinic? (eg. Mobile app, telehealth etc.)
9. Do you recommend developing a digital strategy (e.g. Mobile app, website) that helps you to access SRH information easily during and after the pandemic?

**Ending Questions**

1. Do you have in mind any digital strategies you think the health care system could use to improve access to SRH services for adolescents during and after the pandemic?
2. Is there anything that you might not have thought about before that occurred to you during this interview?
3. Is there anything else you think I should know to better understand about adolescents SRH information needs during the pandemic?
4. Is there anything you would like to ask me?

**Note:** This interview guide will be modified based on input from Adolescent Advisory groups
